# Supplementary material for: Clamping of DNA shuts the condensin neck gate
Source: Proc Natl Acad Sci U S A. 2022 Mar 29;119(14):e2120006119. doi: 10.1073/pnas.2120006119 (PMC9168836; doi:10.1073/pnas.2120006119)
Supplement: Supplementary File [file pnas.2120006119.sapp.pdf]

PNAS
www.pnas.org

Supplementary Information for

Clamping of DNA shuts the condensin neck gate

Byung-Gil Lee*, James Rhodes* and Jan Löwe

Corresponding author: Jan Löwe, email jyl@mrc-lmb.cam.ac.uk

This PDF file includes:

Figures S1 to S7
Table S1

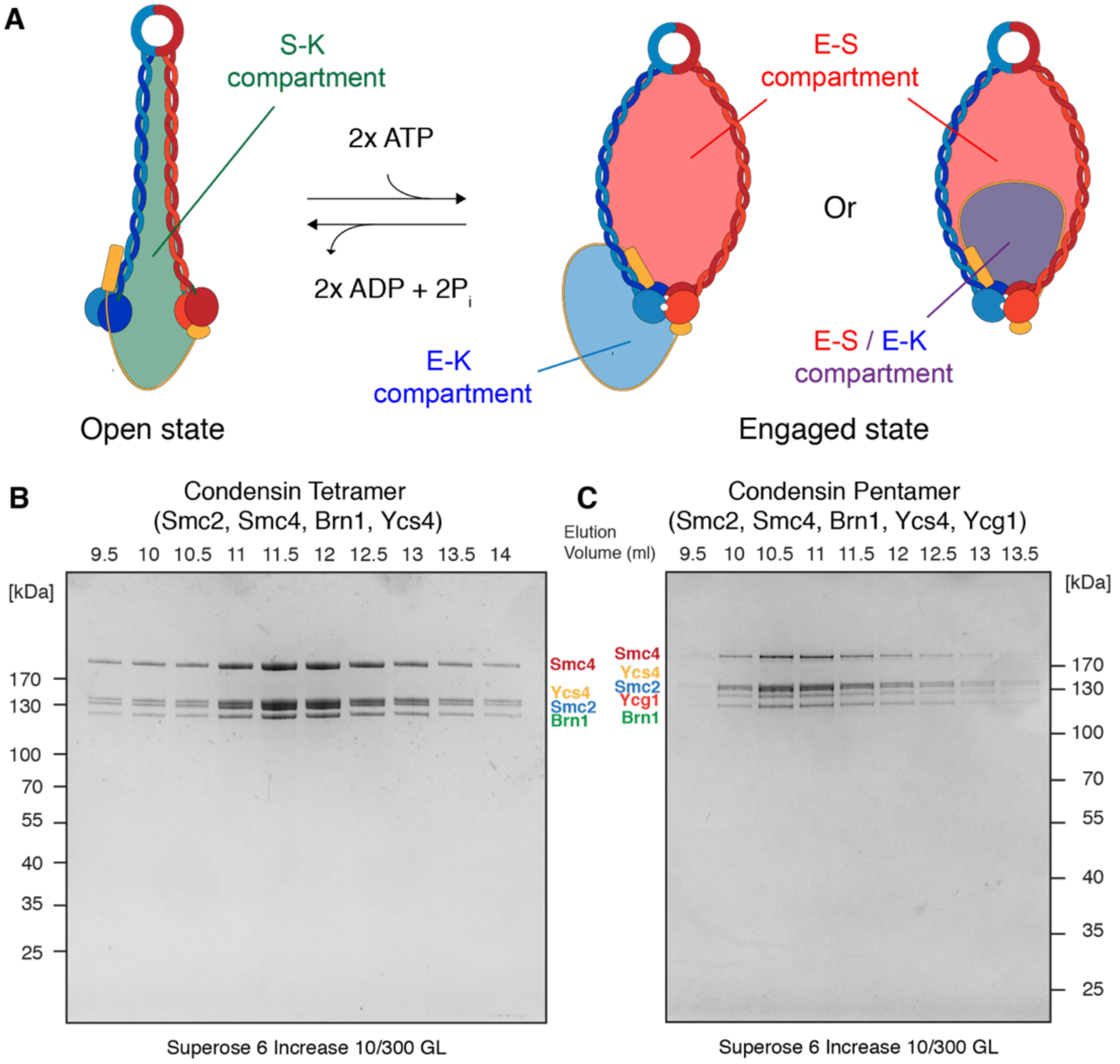

Figure S1. A) Topological compartments of SMC complexes. In condensin, the tripartite Smc2-Smc4-Brn1 ring creates the SMC-Kleisin compartment, S-K. Engagement of the ATPase head domains of Smc2/4 creates two further compartments, Engaged-Kleisin, E-K and Engaged-SMC, E-S. Depending on where the kleisin chain is located, the E-S and E-K compartments can be traversed by the same DNA (right), or not (middle). B) SDS-PAGE gel of the final size-exclusion chromatography of the condensin "tetramer" sample used in this study, comprising subunits Smc2, Smc4, kleisin Brn1 and Ycs4. Elution volumes are provided in mL. C) The same for the condensin "pentamer" sample, comprising Smc2, Smc4, Brn1, Ycs4 and Ycg1.

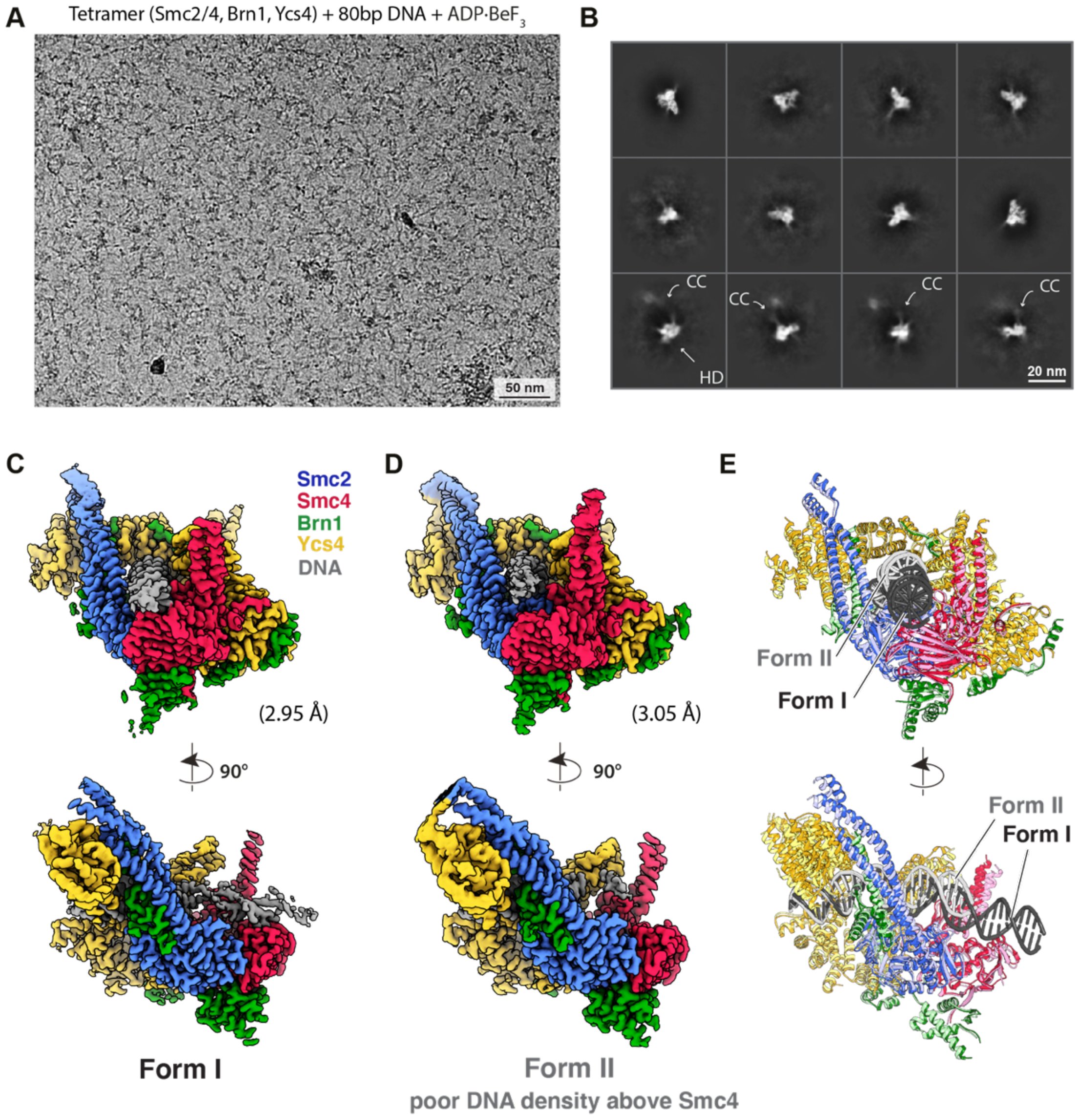

Figure S2. A) Representative micrograph of the cryo-EM dataset used to reconstruct the maps for condensin tetramer in the clamped Forms I and II, after adding 80 bp DNA and ADP.BeF$_3$, and vitrification. B) A selection of 2D class averages of particles picked from A. The head module is well-resolved and shows secondary structure features (two top rows). The bottom row averages show that most of the coiled coil arms and the hinge domain are flexible with respect to the position of the head module. C) Colour-coded cryo-EM map of the condensin tetramer clamped head module. D) The same for Form II. E) Superposition of the refined atomic models for Form I and II, highlighting the only major difference, the length of the DNA as it extends out of the Smc2-Smc4-Ycs4 clamp above the head domains.

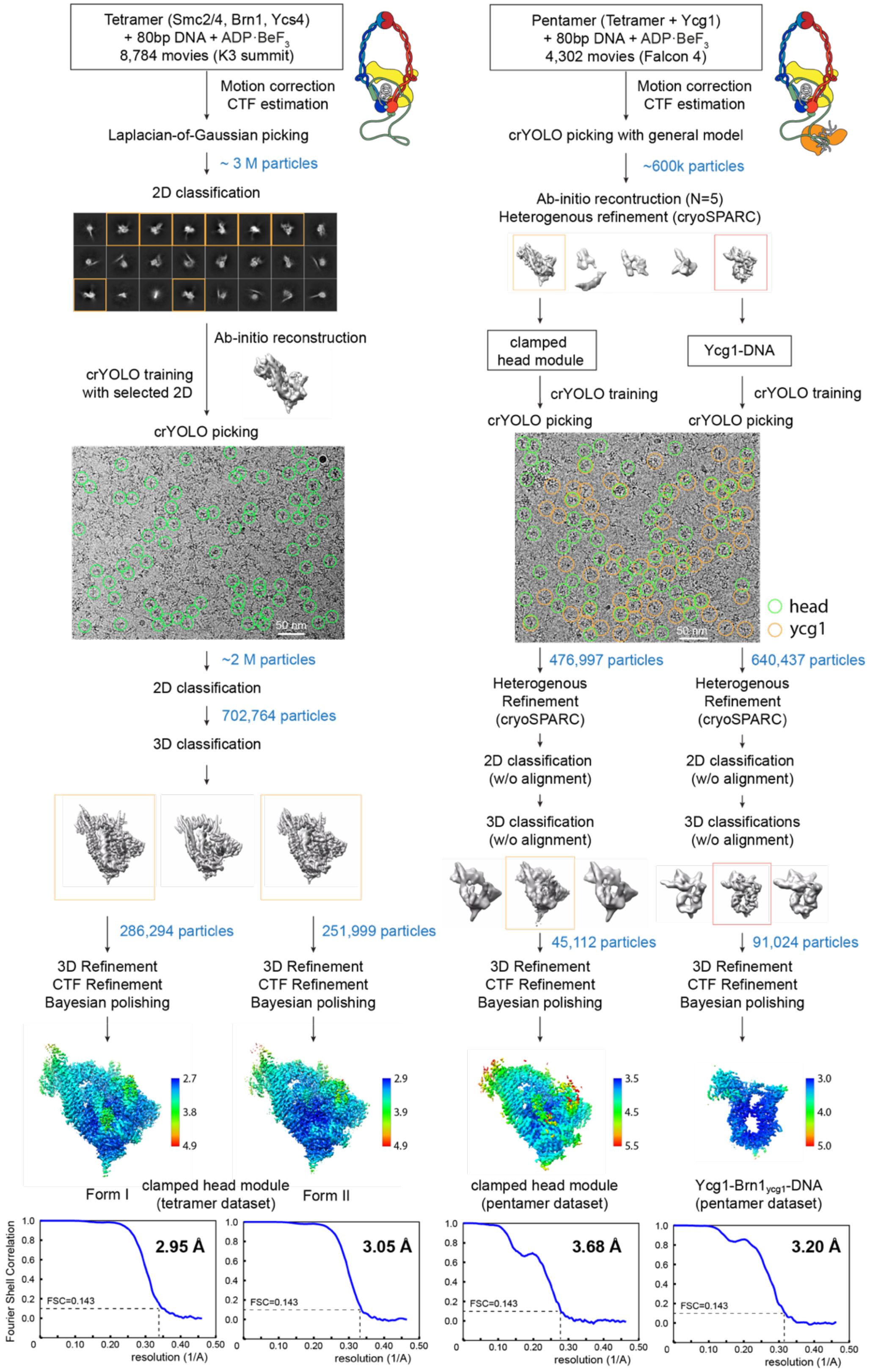

Figure S3. Cryo-EM data analysis and classification workflow. RELION was used unless otherwise specified.

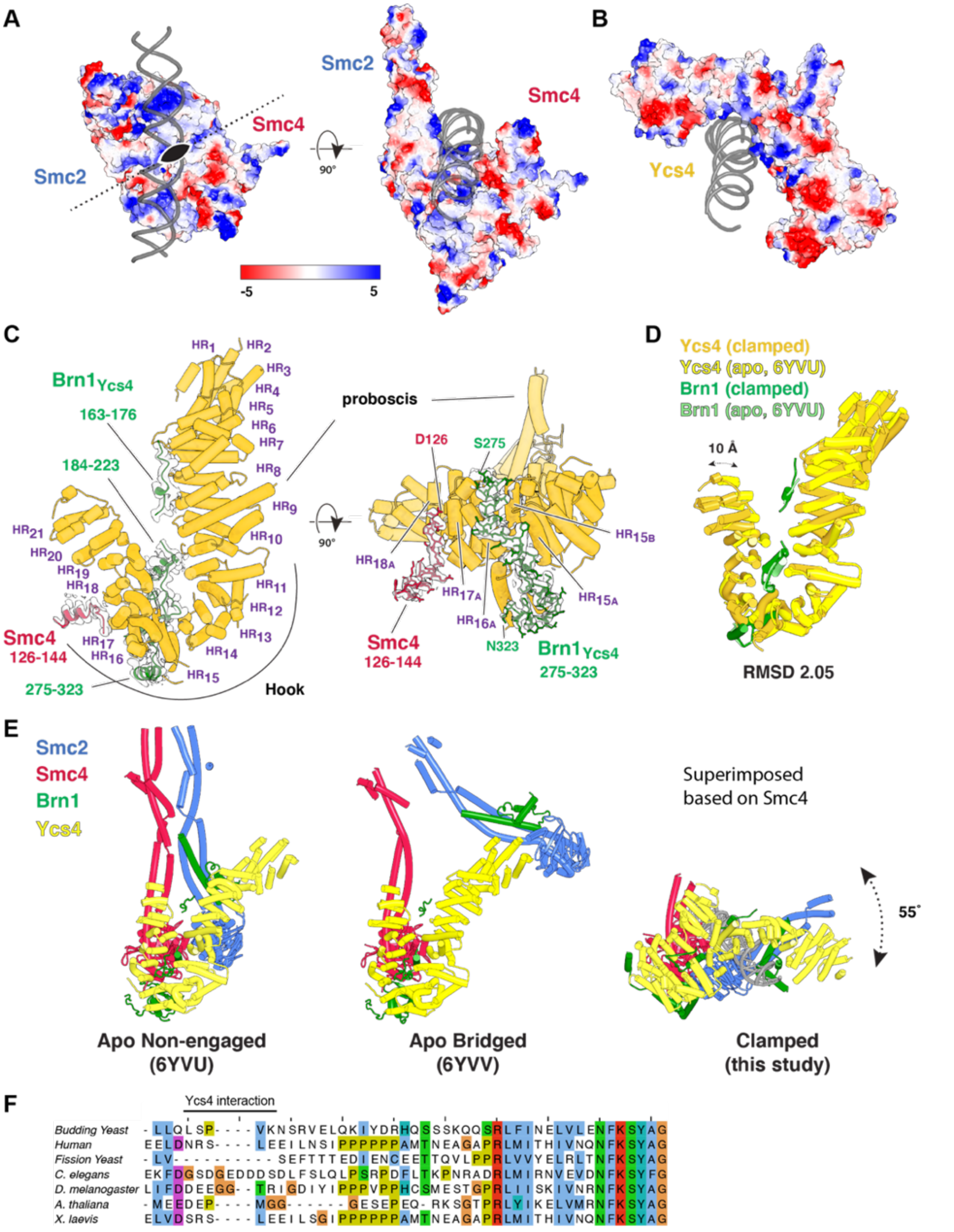

Figure S4. A) Electrostatic potential prediction as calculated in PyMOL, showing positive patches on top of the Smc2/4 head domain heterodimer that coincide with DNA backbone binding. B) The same for Ycs4 as part of the clamp. C) Overview of the HEAT repeat architecture of Ycs4 and showing the cryo-EM map and model of Brn1 sections binding to it in the context of the condensin

tetramer clamp. A section without secondary structure near the N-terminus of Smc4 also interacts with Ycs4 (see also Fig. 2D and H). D) Superposition of the apo condensin Ycs4 structure (PDB 6YVU) (7) and Ycs4 as part of the DNA clamp (this study, Form I). A region near the N-terminus moves by up to 10 Å. E) Comparison of condensin tetramer head module structures in the apo (PDB 6YVU), apo-bridged (6YVV) (7) and DNA clamped (this study, Form I) conformations. Superpositions were done on the head domain of (red) Smc4. Note the models are rotated by 180° around the y-axis relative to Fig. 1C and D (view from the back). F) Multiple sequence alignment of a region of Smc4 (123-169) that includes the Ycs4-interacting region.

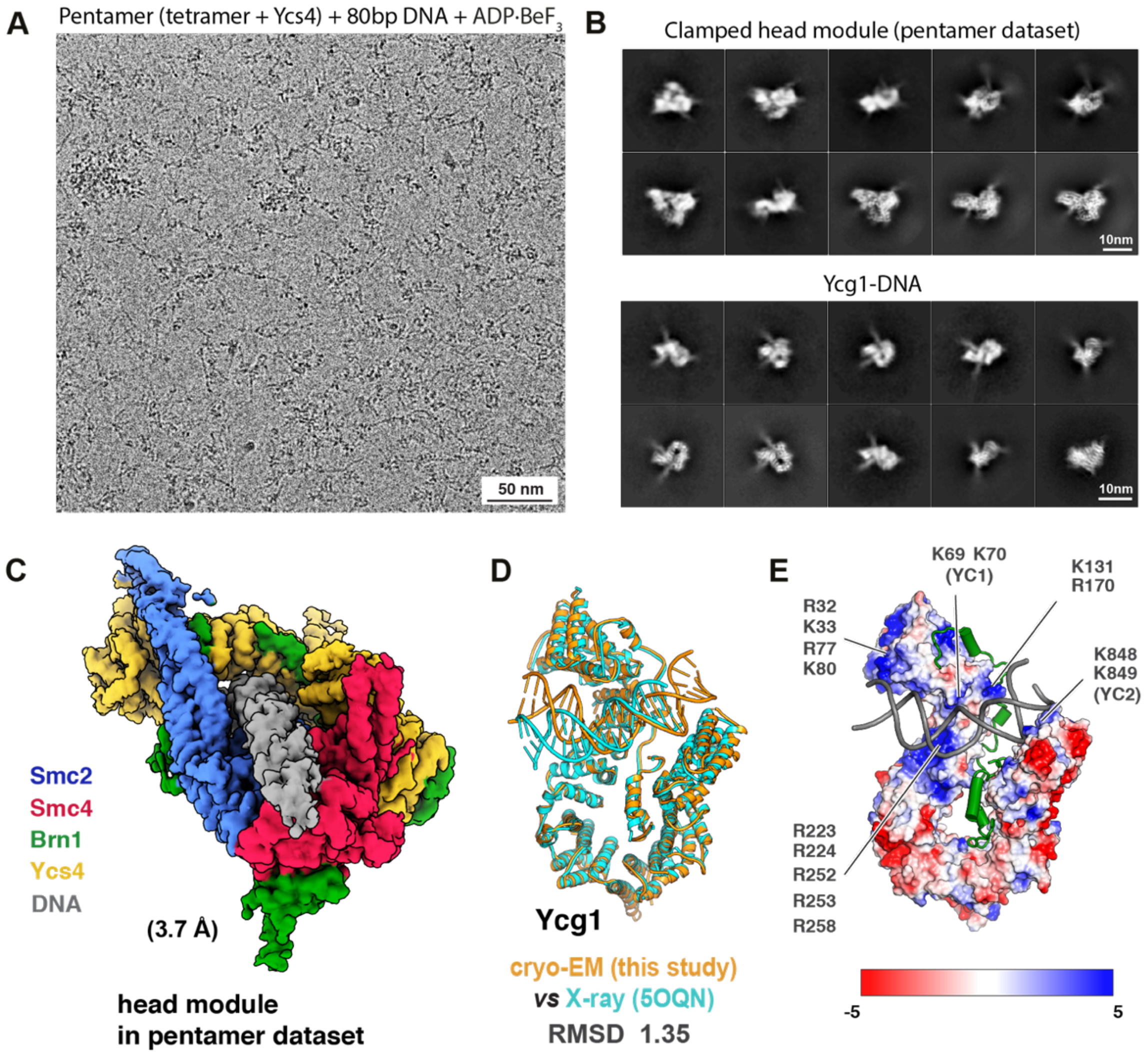

Figure S5. A) Representative micrograph of the cryo-EM dataset used to reconstruct the maps for condensin pentamer in the clamped state, after adding 80 bp DNA and ADP.BeF_3, and vitrification. B) Top: a selection of 2D class averages of particles picked from A, showing the condensin head module. Bottom: a selection of 2D class averages of particles also picked from A, showing Ycg1 bound to DNA. C) Colour-coded cryo-EM map of the condensin pentamer clamped head module, which closely resembles the tetramer structure (Fig. 1C and S2C and D). D) Superposition of a previous X-ray structure of Ycg1 bound to Brn1 and DNA (PDB 5OQN) (19) and Ycg1-DNA as determined here from the condensin pentamer sample. Significant differences in the way the DNA is bound become apparent. E) Electrostatic potential prediction as calculated in PyMOL, plotted onto the surface of Ycg1. Significant positively charged patches are revealed that coincide with DNA backbone binding, including in regions that the X-ray structure did not implicate in DNA binding (the positions YC1 and YC2, which were found in previous work are indicated).

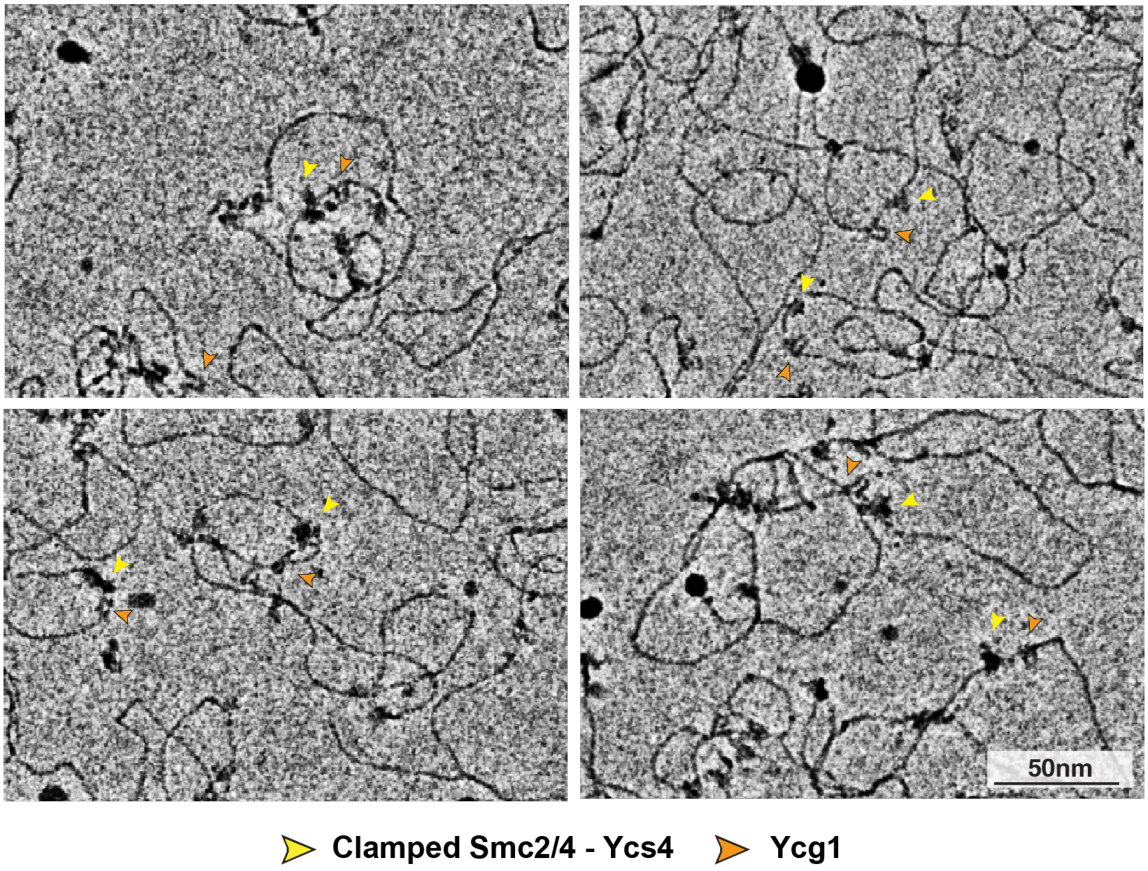

Figure S6. Condensin pentamer clamping circular plasmid DNA, as observed by cryo-EM with a Volta phase plate (VPP), providing more examples of the data shown in Figure 4A. Ycg1 bound to DNA is at a distance from the head module, presumably because Ycg1 is flexibly attached via Brn1.

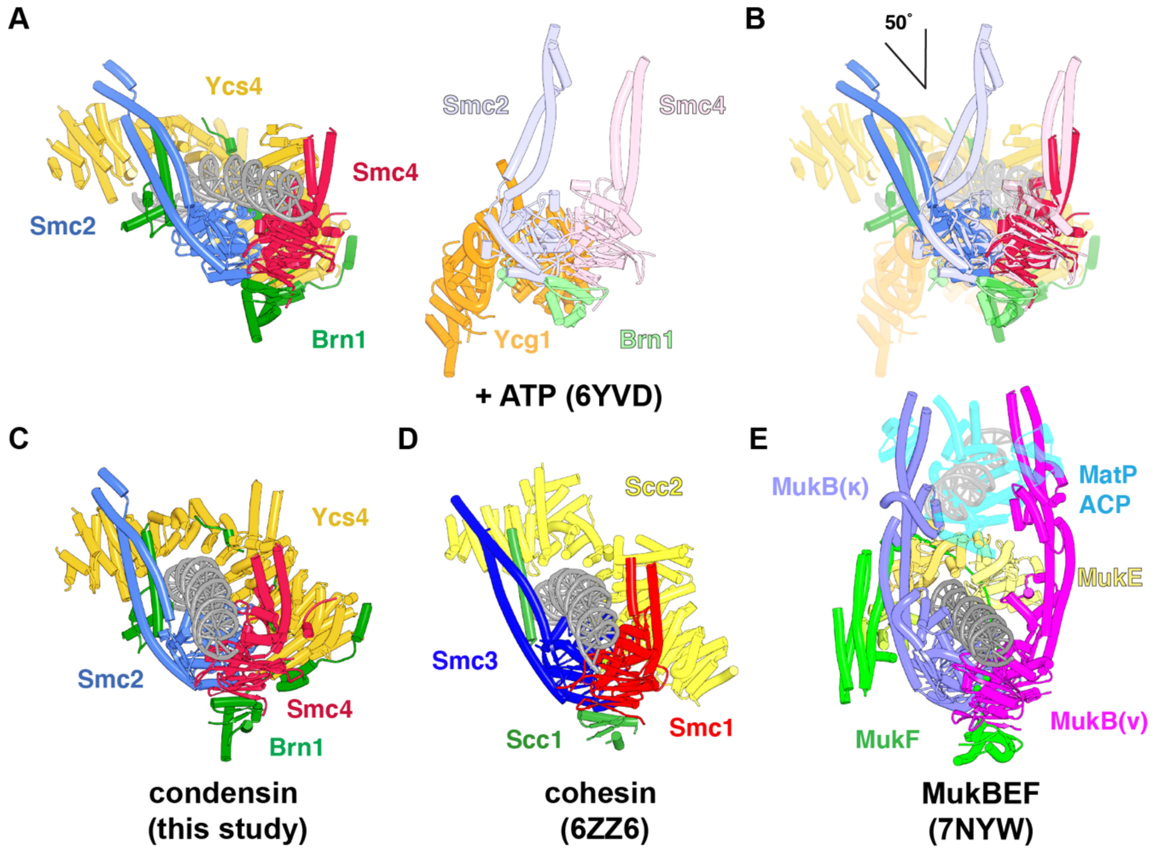

Figure S7. A) Comparison of the DNA-clamped (this study) and DNA-free condensin tetramer structures (PDB 6YVD) (7) in the triphosphate, head-engaged states. B) Superposition of A (centred on red/pink Smc4), showing very significant changes in the position of the coiled coil arms of (blue) Smc2 (open in the clamped state, closed by 50° in the apo engaged state). Ycs4 binds to the head module in the clamped state, whereas Ycg1 occupies a similar but rotated position in the ATP-engaged conformation. C, D and E) Comparison of the clamped states of condensin (this study), cohesin (PDB 6ZZ6) (11, 16, 17) and MukBEF (PDB 7NYW) (14). Condensin and cohesin form very similar clamp structures, with Ycs4 and Scc2 creating the same clamp architectures and with the coiled coil arms open. Note that cohesin's coiled coils remain zipped up in the elbow and hinge-proximal regions further up, at least in some of the particles, despite the coiled coil opening near the heads (11). MukBEF forms an analogous clamp, with the MukE dimer creating the clamp above the DNA. The kleisin MukF could be traced completely in the structure and was shown to remain above the DNA, meaning the DNA is in the E-K compartment. Note the open neck gate between the helical domain of MukF and the neck of kappa-MukB.

Table S1. Cryo-EM data collection, refinement and validation statistics

	Condensin tetramer + 80 bp DNA		Condensin Pentamer + 80 bp DNA		Condensin Pentamer + circular DNA	
	Head Module (Form I)	Head Module (Form II)	Head Module	Ycg1-DNA	Head Module	Ycg1-DNA
**Data collection and processing**						
Microscope and Camera	Titan Krios, K3 summit		Titan Krios, Falcon 4		Titan Krios, Falcon 4	
Magnification	81,000x		75,000x		75,000x	
Voltage (kV)	300		300		300	
No. of micrographs	8,784		4,302		1,016	
No. of micrographs (VPP)	---		---		3,179	
Electron exposure (e⁻/Å²)	55		40		32	
Defocus range (μm)	1.5 ~ 3.3		1.5 ~ 3.0		1.5 ~ 3.0<br>0.5 ~ 0.9 (VPP)	
Physical pixel size (Å)	1.07		1.08		1.08	
Symmetry imposed	*C1*	*C1*	*C1*	*C1*	*C1*	*C1*
Initial particle images (no.)	2,130,610	2,130,610	476.997	694.129	105,614	105,614
Final particle images (no.)	286,794	251,999	45,112	91,024	36,588	27,040
Map resolution (Å)	2.97	3.03	3.68	3.2	8.75	9.0
FSC threshold	0.143	0.143	0.143	0.143	0.143	0.143
Map resolution range (Å)	2.6 – 50	2.6 – 50	3.7 – 50	3.0 – 50	8.75 – 50	9 – 50
**Refinement**						
Initial model used (PDB code)	Apo-condensin (6YVU)	Apo-condensin (6YVU)	---	Ycg1 crystal structure (5OQQ)	---	---
Model resolution (Å)	3.0	3.0		3.2		
FSC threshold	0.143	0.143		0.143		
Map sharpening *B* factor (Å²)	-52	-57		-39.4		
Model composition						
Non-hydrogen atoms	18,240	17,755		8,258		
Protein residues	2,098	2,076		902		
Nucleotide residues	72	56		46		
Ligands	2 ADP<br>2 BeF_3<br>2 Mg	2 ADP<br>2 BeF_3<br>2 Mg		---		
B factors (Å²)						
Protein	30.28	62.79		34.99		
Nucleotide	97.07	150.04		89.42		
Ligand	22.26	54.54		---		
R.m.s. deviations						
Bond lengths (Å)	0.002	0.002		0.002		
Bond angles (°)	0.526	0.592		0.482		
Validation						
MolProbity score	1.53	1.71		1.63		
Clashscore	7.66	8.57		4.20		
Poor rotamers (%)	0.00	0.00		0.00		
Ramachandran plot						
Favored (%)	97.42	96.31		93.36		
Allowed (%)	2.58	3.64		6.53		
Disallowed (%)	0.00	0.05		0.11		
PDB ID	**7Q2X**	**7Q2Y**	---	**7Q2Z**	---	---
EMDB ID	**13783**	**13784**	**13785**	**13786**	**13787**	**13788**
